# Application of Microwave Ablation for Treating Pulmonary Adenocarcinoma: A Case Report

**Published:** 2017-06

**Authors:** Arda Kiani, Kimia Taghavi, Hadi Rokni-Yazdi, Atefeh Abedini

**Affiliations:** 1 Tracheal Diseases Research Center, National Research Institute of Tuberculosis and Lung Diseases (NRITLD), Shahid Beheshti University of Medical Sciences, Tehran, Iran,; 2 Chronic Respiratory Diseases Research Center, NRITLD, Shahid Beheshti University of Medical Sciences, Tehran, Iran,; 3 Department of Radiology and Imaging, MIC, Imam Khomeini Hospital, Tehran University of Medical Science, Tehran, Iran.

**Keywords:** Microwave tumor ablation, Adenocarcinoma, Microwave

## Abstract

Pulmonary metastases occasionally do not respond to chemotherapy and surgery. Moreover, some early stage cancer patients, who are candidates for surgery, are unable to tolerate surgery. In such cases, microwave ablation is a convenient alternative for tumor eradication. Microwave ablation has low morbidity and mortality rates compared to traditional cancer treatments. Moreover, the lower procedural cost, potential to perform treatment in an outpatient setting, and repeatability are beneficial aspects of this technique. Herein, we report an adenocarcinoma case that was potentially unsuitable for surgery and was treated with percutaneous microwave pulmonary tumor ablation in August 2015 at the Bronchoscopy and Laser ward of the Shahid Beheshti Medical University, Iran.

## INTRODUCTION

Cryotherapy and heat therapy methods are medical techniques prevalent in Iran. In the Laser and Bronchoscopy ward of the Masih Daneshvari hospital, cryotherapy has being successfully used for the treatment of papillomatous lesions for over a decade now. In addition, the surgical team of the hospital has been utilizing heat therapy methods such as laser and argon plasma coagulation over the last 25 years.

Heat ablative therapies have been used for the purpose of tumor eradication for a long time. Such methods are applied for mediastinal and hilar lung tumors, including the tracheobronchial tree and for tumors attached to the chest, where bronchoscopy is not a viable option, and surgery or chemotherapy are the only remedies. Pulmonary metastases occasionally do not respond to chemotherapy and surgery. Moreover, some early stage cancer patients, who are candidates for surgery, are unable to tolerate surgery. In such cases, radiofrequency (RF) ablation and microwave tumor ablation are safe and effective alternatives. Moreover, microwave tumor ablation has been previously used as a treatment option for liver and bone cancer (1).

Microwave tumor ablation is defined as the direct application of thermal therapies to achieve tumor destruction. This method is a new approach in the treatment of cardiac arrhythmia, tumors, bone metastases, and endometrial disorders. Electromagnetic microwaves are radiated directly onto localized tissue, leading to agitation of water molecules in the surrounding tissue, thereby producing friction and high temperature (approximately 150°C), thus inducing cellular death via coagulation necrosis. Microwave tumor ablation therapy is associated with low morbidity and mortality rates compared to traditional cancer treatments. Moreover, lower procedural cost, the potential to perform ablation in an outpatient setting, and repeatability are the beneficial aspects of this treatment method (2,3). Herein, we report an adenocarcinoma case that was potentially unsuitable for surgical procedures but was treated with percutaneous microwave pulmonary tumor ablation in August 2015, at the Bronchoscopy and Laser ward of the Shahid Beheshti Medical University, Iran.

## CASE SUMMARIES

A 75-year-old man was clinically examined by an interventional pulmonologist, prior to microwave ablation. The medical history and physical examination details were documented. All index tumors were measured in three dimensions. The 68- × 48-mm pulmonary tumor arising from the pleura and close to the aorta was examined using computed tomography (CT) and magnetic resonance imaging, and biopsy confirmed the final diagnosis to be malignant adenocarcinoma. The patient received standard treatment for tumor control. However, standard treatments failed and the tumor kept increasing in size despite chemotherapy. Alternative treatment options were discussed with the patient; the patient indicated a preference for microwave ablation, following which, the risks and benefits of the procedure, including pneumothorax, fistula, and bleeding, were explained. The review board of the Shahid Beheshti Medical University approved the current treatment. Complete blood cell count, platelet count, and coagulation test results were obtained. The patient was asked to stop anticoagulant and antiplatelet medications 2–7 days prior to ablation. The patient presented at the laser ward on the day of the procedure after a 12-hour fast. Informed consent was obtained from the patient. Standard surgical preparations were performed. Local anesthesia was attained via intradermal and intravenous injection of 1% lidocaine hydrochloride solution. A bispectral index monitor was used to maintain it at 50–60 throughout the procedure. Continuous pulse oximetric, electrocardiographic, and blood pressure monitoring was carried out at 5-minute intervals. CT-guided percutaneous microwave ablation of the pulmonary masses was carried out in the patient in one session. Ablation was performed with CT fluoroscopic guidance. The tumor was localized using CT and an optimal intraparenchymal approach to the left upper side of the left lung was finalized.

**Figure 1.**
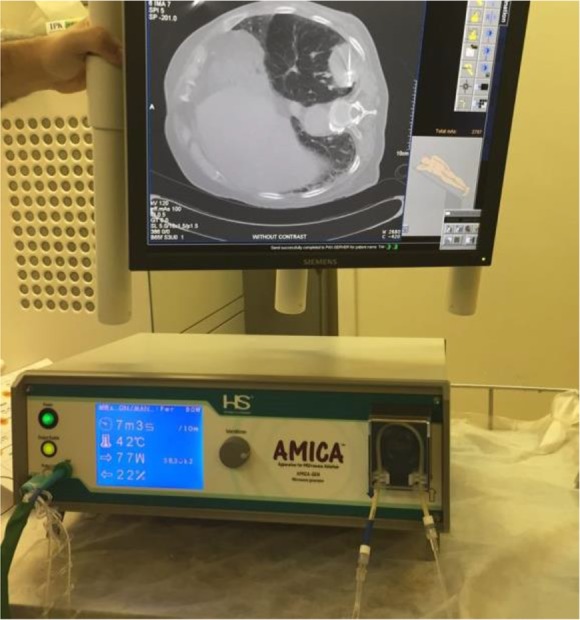
Post treatment CT image demonstrates a biopsy-proved 5-cm adenocarcinoma tumor of upper lobe of left lung

The procedure was performed by board-certified pulmonologists, a board-certified interventional radiologist, and specialized nurses according to previous guidelines (4,5). The utilized microwave generator (HS Amica-Gen; HS Hospital Service, Rome, Italy) was able to generate up to 80 W of power at a frequency of 915 MHz. After creating a small incision in the skin, a thin microwave antenna was guided across the lung, from the opposing side of the tumor, and the probe was placed at the terminal end of the tumor. According to the manufacturer’s instructions, 80-W radiation was applied for 10 minutes. According to previous studies in vitro, we assumed the intratumoral temperature in the current case will reach 180 ° C (5).The body probe temperature reached 45°C, which was cooled down by perfusing the outer shaft of the antenna using a water peristaltic pump. After ablation, the patient was transferred to the radiology recovery room. As the surgery site was near the pleura and intercostal nerves, a pain pump was instilled to prevent pain. No bronchopleural fistula was observed on initial chest radiography, and a 22- × 3-cm loculated pneumothorax was noted. Post procedural 2-hour chest radiography showed no enlargement of the pneumothorax, and therefore, dilatory pneumothorax was excluded as a possible diagnosis. The vital signs were monitored at regular intervals. The patient showed normal blood pressure and overall health status without any signs of serious complications. Mild muscle pain and slight fever of the patient decreased after 48 hours of admission to the general ward and the patient was discharged. Post-ablation CT examinations at 3 months after ablation showed no recurrence in the lungs. However, 6 months later, the patient developed brain tumor metastasis and died of cerebral complications.

**Figure 2.**
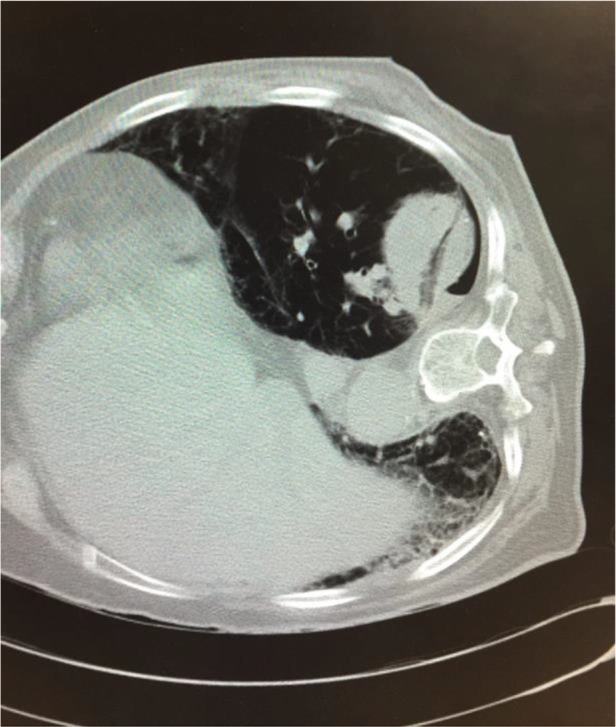
Post ablations image with 1 antenna powered by a 915 MHz generator delivering a total of 80 W for 10 minutes. The line represents the ablation zone created through the ablation

### Technical review

Microwave ablation refers to the application of electromagnetic microwaves with a short wavelength of 915 MHz to 2.45 GHz (4,5). The electromagnetic spectrum between infrared radiation and radio waves, comprising frequencies between 900 and 2450 MHz, refers to microwave radiation. The 9.2 × 108 Hz microwave radiation has an electrical charge with about two billion oscillation per second between positive and negative electro charges (1). Water is the highest component of biological tissues. Two characteristics of the dielectric hysteria and the bipolar momentum in asymmetric dipole molecules, such as water, cause the water molecules to move along the microwave magnetic field. (3). Water molecules do not move as fast as the magnetic field, which results in some energy being converted into heat. The increased kinetic energy is the outcome of continual realignment, which results in elevated temperatures. Microwaves penetrate human tissues at temperatures between 60°C and 100°C, resulting in prompt coagulation of tissues and irreversible damage to DNA and mitochondrial and cytosolic enzymes of the cells. Tissues carbonize and vaporize at more than 100–110°C (6).

The conductivity of tissue determines how radiation is absorbed by the tissue. Effective conductivity is dependent essentially on the type of tissue and the frequency of the applied field. Isotropic dielectric properties lead to almost equivalent effective conductivity in all types of tissues, excluding muscles (1,7).

The heating rate relates proportionally to conductivity, but the penetration depth relates inversely to conductivity. Deeper penetration occurs on slower heating (7). The treatment of tumors would be more effective due to several advantages of microwave ablation compared to similar heat treatments such as RF ablation. Some of the benefits of microwave ablation include: larger tumor ablation volumes, higher intratumoral temperatures, potency of using multiple applicators, the presence of complementary ventilation system, shorter ablation time, less procedural pain and optimum heating to cystic masses (1).

The most important distinct property of microwave energy is propagation through non-metallic materials and all types of tissues, including water vapor or dehydrated water (3). Microwaves also offer more direct heating than RF, making it more effective in organs with high blood perfusion and causing less damage to the surrounding tissues (3). Therefore, despite the availability of RF ablation in the current case, we preferred to use microwave ablation. Microwaves have also been shown to offer suitable wavelength and tissue penetration depth in several medical applications. Wave penetration is approximately 2–4 cm in most tissues, at 915 MHz to 2.45 GHz, which is frequently comparable to treatment targets such as 2–4-cm tumors (3).

### Clinical applications of microwave ablation

#### Liver Tumors

Mild 60-watt microwave ablation was previously shown to have comparable effectiveness and 3-year survival rates, to standard treatments for small hepatocellular carcinomas. In contrast, microwave ablation seems to be ineffective for large tumors (>3 cm) such as colorectal hepatic metastatic disease. In addition, owing to the benefit of treating multiple lesions through one ablation schedule, microwave ablation has advantages for both primary and metastatic liver cancer treatment (8,9).

#### Prostate Tumors

Although prostate tumors were among the first tumors for which microwave ablation was used for treatment, currently, microwave ablation is less frequently used, in order to prevent the probable risk of destruction to nerves (9).

#### Bone Malignancies

The low conductivity of bones limits the effectiveness of RF ablation due to thermal conduction. In contrast, microwaves can provide deeper penetration with higher impedance. In both primary and metastatic bone cancers, relative advantage of microwave ablation is evident. In this setting, a relative advantage for microwave ablation is evident. Furthermore, microwaves do not seem to cause tissue heating and dehydration, which provides opportunities for more effective heating and deeper penetration based on the permittivity of the respective bone (8).

#### Kidney Tumors

The kidney as a vascular organ with high amounts of central perfusion that creates a notable heat sink. Previous studies indicate microwave ablation as an effective treatment for renal cell carcinoma. Although these initial findings are promising, further studies of kidney tumors are mandatory (8,9).

#### Lung Tumors

Poor thermal conduction and low electrical conductivity are observed in aerated lungs, limiting the potency of RF energy. Microwaves, on the other hand, may have notable advantages in the lungs. The microwave heating volume does not decrease in the presence of high impedance and low conductivity of aerated lungs. In fact, deeper microwave penetration is observed in the lungs compared to the liver. To the best of our knowledge, no clinical trials have demonstrated microwave ablation as an effective treatment for lung tumors (8,9). Large tumors may require two to 13 antennas for proper ablation. Cells destroyed by microwaves are gradually eliminated but will be replaced with scar tissue and fibrosis (6). Local recurrence generally occurs at the edge of the tumor, and in such cases, treatment should be repeated (6). Pleural effusion is a common complication after microwave ablation for lung tumors. Although microwave ablation is suggested to be safe and effective for the treatment of lung tumors, further studies are recommended.

Microwave tumor ablation has also been used in relieving pain associated with metastasis and angioplasty, in addition to the treatment of cancer, cardiac arrhythmias, and endometrial disorders (8, 10).

## CONCLUSION

In the current study, microwave ablation was performed via an antenna that was guided from the side opposite to the tumor. New guidelines recommend the direct entry of the antenna in the tumor, to prevent probable pleural fistula. Current studies indicate that microwave ablation is a safe treatment modality for pulmonary malignancies and that it improves survival of patients unsuitable for surgery.
